# Key Role for Clumping Factor B in Staphylococcus aureus Nasal Colonization of Humans

**DOI:** 10.1371/journal.pmed.0050017

**Published:** 2008-01-15

**Authors:** Heiman F. L Wertheim, Evelyn Walsh, Roos Choudhurry, Damian C Melles, Hélène A. M Boelens, Helen Miajlovic, Henri A Verbrugh, Timothy Foster, Alex van Belkum

**Affiliations:** 1 Erasmus MC, University Medical Center Rotterdam, Department of Medical Microbiology and Infectious Diseases, Rotterdam, The Netherlands; 2 Moyne Institute of Preventive Medicine, Microbiology Department, Trinity College, Dublin, Ireland; University of California San Francisco, United States of America

## Abstract

**Background:**

Staphylococcus aureus permanently colonizes the vestibulum nasi of one-fifth of the human population, which is a risk factor for autoinfection. The precise mechanisms whereby S. aureus colonizes the nose are still unknown. The staphylococcal cell-wall protein clumping factor B (ClfB) promotes adhesion to squamous epithelial cells in vitro and might be a physiologically relevant colonization factor.

**Methods and Findings:**

We define the role of the staphylococcal cytokeratin-binding protein ClfB in the colonization process by artificial inoculation of human volunteers with a wild-type strain and its single locus *ClfB* knock-out mutant. The wild-type strain adhered to immobilized recombinant human cytokeratin 10 (CK10) in a dose-dependent manner, whereas the *ClfB^−^* mutant did not. The wild-type strain, when grown to the stationary phase in a poor growth medium, adhered better to CK10, than when the same strain was grown in a nutrient-rich environment. Nasal cultures show that the mutant strain is eliminated from the nares significantly faster than the wild-type strain, with a median of 3 ± 1 d versus 7 ± 4 d (*p* = 0.006). Furthermore, the wild-type strain was still present in the nares of 3/16 volunteers at the end of follow-up, and the mutant strain was not.

**Conclusions:**

The human colonization model, in combination with in vitro data, shows that the ClfB protein is a major determinant of nasal-persistent S. aureus carriage and is a candidate target molecule for decolonization strategies.

## Abstract 2

Heiman Wertheim and colleagues investigate the role of*Staphylococcus aureus* clumping factor B, a cell wall protein, in bacterial adherence to epithelial cells and persistent colonization of human nostrils.

## Editors' Summary

**Background.:**

Staphylococcus aureus are common bacteria that normally live on the skin. They also colonize the nostrils of about one in five adults permanently and another one in three adults intermittently. Although these bacteria usually coexist peacefully with their human carriers, they can cause minor infections such as pimples and boils if they enter the skin through a cut or a sore. They can also cause potentially life-threatening infections such as blood poisoning and pneumonia. These serious, invasive infections are often “autoinfections.” That is, they are caused by strains of S. aureus that are present in the patient's nose before they become ill. Minor S. aureus infections can be treated without antibiotics—by draining a boil, for example. Invasive infections are usually treated with antibiotics such as flucloxacillin.

**Why Was This Study Done?:**

There is no effective vaccine against S. aureus infections and these bacteria are becoming increasingly resistant to flucloxacillin, methicillin, and other antibiotics. Worryingly, although methicillin-resistant S. aureus (MRSA) infections occur most frequently among people in health-care facilities who have weakened immune systems, community-acquired MRSA infections among otherwise healthy people are increasingly common. Consequently, new ways to avoid S. aureus infections are urgently needed. Because persistent nasal carriers of S. aureus have an increased risk of infection, one strategy might be to prevent nasal colonization with S. aureus. How these bacteria colonize the nose is poorly understood, but is likely to involve interactions between molecules expressed on the surface of the bacteria and molecules expressed on the surface of the cells lining the nostrils. In this study, the researchers use a new human nasal colonization assay to investigate the involvement of a bacterial surface protein called clumping factor B (ClfB) in the survival of S. aureus in the human nose. ClfB binds to cytokeratin 10, a protein expressed by cells lining the human nose, and has been implicated in the colonization of mouse noses by S. aureus.

**What Did the Researchers Do and Find?:**

The researchers introduced a strain of S. aureus that made ClfB and an otherwise identical, mutant strain that lacked ClfB into the nostrils of healthy human volunteers and measured how long the two strains survived. For safety reasons, the S. aureus strains used in this study have an additional defect that makes them less likely to colonize and persist in the human nose than the strains found in natural S. aureus carriers. Although both strains grew equally well in the laboratory, the mutant strain was eliminated from human noses much quicker than the strain that made ClfB. Mutant bacteria lacking ClfB were cleared from the nostrils of all the volunteers within two weeks, whereas the bacteria that made ClfB were still present in some of the volunteers four weeks after their introduction. When the researchers investigated how well the two strains stuck to a layer of human cytokeratin 10 in a plastic dish, they found that the bacteria that made ClfB stuck to the human protein but the mutant bacteria did not. Furthermore, the strain with ClfB stuck particularly well to cytokeratin 10 when the bacteria had been grown in conditions where nutrients were limiting, a situation that mimics bacterial growth in the human nose.

**What Do These Findings Mean?:**

These findings show that ClfB is an important factor in the establishment of human nasal colonization by S. aureus and suggest that ClfB might be a target for S. aureus decolonization strategies. Furthermore, although ClfB is clearly important in human nasal colonization by S. aureus, it is likely that additional bacterial factors will also be involved in this process. The human nasal colonization model used in this study may be useful in the identification of these additional factors and also as a test bed for potential S. aureus decolonization strategies.

**Additional Information.:**

Please access these Web sites via the online version of this summary at http://dx.doi.org/10.1371/journal.pmed.0050017.

- The MedlinePlus encyclopedia has a page on *Staphylococcus aureus* and MRSA (in English and Spanish)
- The US Centers for Disease Control and Prevention provides information on community-associated MRSA (in English and Spanish)
- The UK National Health Service's health website (NHS Direct) provides information about staphylococcal infections and about MRSA
- The UK Health Protection Agency provides information about Staphylococcus aureus

## Introduction

Staphylococcus aureus remains one of the prime human bacterial pathogens, associated with significant morbidity and mortality worldwide. The combination of an increasing number of antimicrobials to which this pathogen has become resistant and the lack of an effective vaccine underscores that alternatives to combating S. aureus disease are urgently required. In addition, community-acquired infections with methicillin-resistant S. aureus (MRSA) are rising steeply [1,2]. Approximately 80% of invasive S. aureus infections are autologous [3,4] in that they are caused by strains carried in the nose by the patient prior to illness. Approximately 20% of the adult population carries S. aureus in their nose persistently, another 30% intermittently, whereas 50% are non-carriers [5].

Persistent nasal carriers of S. aureus have an increased risk of infection. Therefore, discovering strategies that prevent nasal colonization of S. aureus in patients at risk of (auto)infection is attracting increasing attention. Intervening in S. aureus colonization, however, requires the identification of human and bacterial factors that are important in the process [6,7].

Nasal colonization by S. aureus is likely to be facilitated by its surface structures. These range from peptidoglycan molecules and cell-wall teichoic acids to a wide variety of proteins belonging to the family of microbial surface components recognizing adhesive matrix molecules (MSCRAMMs) [8,9]. The MSCRAMM clumping factor B (ClfB) was recently identified as a major determinant of nasal colonization of the mouse [10,11]. ClfB binds to human-type I cytokeratin 10 (CK10), which is expressed on squamous epithelial cells [12]. In addition, it was shown that anti-ClfB antibodies could actively and passively protect mice from staphylococcal colonization in the nose [13].

However, experiments to prove causal involvement of bacterial factors in maintaining the colonized state were performed in vitro or in animal models of questionable relevance to the human situation [6,14,15]. None of the animal species used in model experiments are natural nasal carriers of S. aureus. Other studies relied on indirect clinical or microbiological observations to define the involvement of bacterial determinants in colonization and disease in humans [16]. To our knowledge, in vivo experiments on the relevance of bacterial factors required for S. aureus nasal colonization in humans have not been performed thus far.

Although animal models may help define the molecular mechanisms leading to colonization of the nose, a human model of colonization will probably be most appropriate [17,18]. Such an experimental model is essential in the design of new strategies to prevent infection. This is important since recent trials with topical antibiotics showed little to no preventive effect on the rate of nosocomial S. aureus infection, due to the poor efficacy of this type of treatment [19–21].

The present study investigates the survival of two different S. aureus strains in a novel human nasal colonization model. We used an S. aureus strain (8325–4) expressing ClfB and its isogenic knockout, deficient in ClfB (DU5997). It was hypothesized that ClfB is essential for adherence to the nasal mucosa, hence absence of this factor would lead to a significant reduction in S. aureus survival in the human nose. Furthermore, we performed in vitro testing to demonstrate that the S. aureus cells growing under in vivo–like conditions can express ClfB and that these bacteria adhere to immobilized CK10.

## Materials and Methods

### Study Population

Sixteen healthy volunteers (six males and ten females, median age 32 y [range 19–51 y]) were included in this study. Prior to enrollment, potential participants received a medical checkup. Volunteers with a medical history of diabetes mellitus, renal insufficiency, chronic skin disease (eczema or psoriasis), chronic obstructive lung disease, disease of the heart valve, immune deficiency, or recent antibiotic use were excluded. Volunteers in close contact with individuals suffering from the afflictions mentioned above were excluded as well. Informed consent was obtained. Participants were notified of the fact that a dedicated infectious disease physician would be on call for the entire study period.

### Ethical Clearance

The study protocol was approved by the Medical Ethical Committee of Erasmus MC (MEC 156.137/1996/186). Permission to inoculate volunteers with genetically modified S. aureus strains was obtained from the Dutch authorities (Dutch Committee on Genetic Modification [COGEM] protocols BGGO 03/02.13 and CGM/040224–04).

### S. aureus Strains

Strains 8325–4 (wild-type) and DU5997 (mutant) were used for artificial nasal colonization of the human volunteers. The genome sequence of the original 8325 strain is available at http://www.genome.ou.edu. The 8325–4 strain differs from 8325 by the absence of three prophages [22]; 8325–4 has a defect in the stationary-phase sigma factor (SigB) [23]. This strain is therefore unlikely to colonize and persist as well as 8325-4 wild-type strain (WT) or DU5997 (mutant strain) and thus facilitated a safe inoculation protocol for humans. DU5997 is a *ClfB^−^* mutant of S. aureus 8325–4 with a promoter-less *tetK* gene (encoding low-level tetracycline resistance) inserted in the *clfB* gene, as described previously [24]. For comparative growth experiments, we additionally used the S. aureus strain SH1000, a derivative of strain 8325–4, which expresses wild-type levels of SigB [25].

### Growth Experiment

To reflect the in vivo situation, bacterial strains were grown in RPMI 1640 (Sigma), a minimal medium which is limited in iron. Strains were also grown in tryptic soy broth (TSB). Starter cultures were diluted in 60 ml of RPMI or TSB to an OD_600_ of 0.1 and grown at 37 °C with shaking (200 rpm) and absorbance at 600 nm monitored for 32 h (RPMI) or 24 h (TSB) using a spectrophotometer.

### Study Design: Artificial Inoculation Protocol

**Figure 1:**
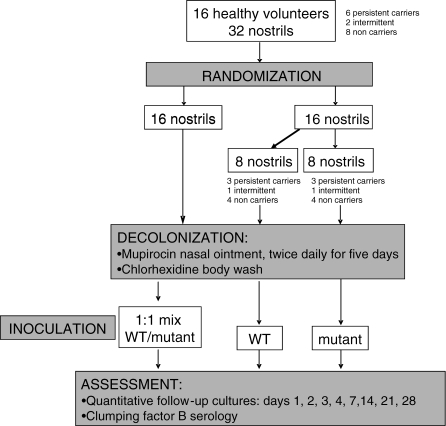
Study Design First, the left or right nostril was randomized to receive the inoculation mix, containing strains 8325-4 (WT) and DU5997 (mutant strain). Second, the contralateral nostril was randomized to receive either the naturally occurring (“WT” in figure) or mutant strain.

From all enrolled volunteers, we obtained two nasal cultures 1 wk apart before artificial inoculation in order to differentiate between persistent, intermittent, and non-carriers, as described previously (Nouwen et al. [26]). To be classified as a persistent carrier, both nasal cultures needed to be positive with a *S. aureus.*. In case of one positive culture, the volunteer was classified as an intermittent carrier. For non-carriers, all cultures needed to be negative. Figure 1 illustrates the design of the study. Decolonization treatment was started for all volunteers (nasal mupirocin twice daily for 5 d in combination with once-daily washing with chlorhexidine-containing soap for 5 d [Hibiscrub; Regent Medical]). Five weeks after mupirocin and chlorhexidine treatment, nostrils were cultured again to assess colonization status, and experimental nasal inoculation was performed.

The two inoculation strains, 8325–4 and DU5997 (*ClfB^−^*), were cultured separately to log phase in TSB. A mixture was prepared immediately prior to inoculation. In one nostril, 2 × 10^7^ cfu of the 8325–4/DU5997 mixture in a 1:1 ratio was applied, whereas in the contralateral nostril, 1 × 10^7^ cfu of either the 8325–4 or the DU5997 were installed. The nostrils of volunteers were randomized as follows: first, the left or right nostril was randomized to receive the inoculation mix by drawing a sealed envelope. Second, another envelope was drawn that contained instructions as to which single strain (8325-4 or DU5997) should be inoculated in the contralateral nostril. Thus, all 16 subjects had one nostril inoculated with the mixed culture, and half had the contralateral nostril inoculated with 8325–4 and the other half with DU5997. Randomization was stratified by carriage status.

This design allowed for the assessment of the survival of both strains, depending on two different inoculation protocols. The inoculation was performed in a blinded fashion to prevent bias in reading the growth results of the nasal cultures. Follow-up cultures were performed on day 1, 2, 3, 4, 7, 14, 21, and 28 after inoculation (Figure 1).

### SDS-PAGE, Western Immunoblotting, and Zymography

S. aureus cells were suspended to an OD_600_ of 40 in 30% raffinose plus 20 mM MgCl_2_ in a final volume of 100 μl. To each sample, 12-μl lysostaphin (2 mg/ml) and 8-μl protease inhibitors (Complete cocktail; Boehringer Mannheim) were added, and the suspension was incubated at 37 °C for 20 min. Protoplasts were removed by centrifugation at 12,000*g* for 10 min. Supernatants containing wall-associated proteins were boiled for 5 min in an equal volume of final sample buffer (0.125 M Tris-HCl [pH 6.8], 4% [w/v] SDS, 20% [v/v] glycerol, 10% [v/v] β-mercaptoethanol, and 0.002% [w/v] bromophenol blue). SDS-PAGE was performed by standard methods. Proteins were electrophoretically transferred to PVDF Western blotting membranes (Boehringer Mannheim) by the wet system (Bio-Rad). Membranes were incubated overnight at 4 °C in 10% blocking reagent (Marvel milk powder). Primary anti-ClfB A region antibody was used at a dilution of 1:5,000 for a 1-h incubation at room temperature. Protein A–conjugated horseradish peroxidase (Sigma, a 1 mg/ml stock diluted 1:500) was used to detect bound antibody by incubation for 1 h at room temperature. Membranes were developed using LumiGLO chemiluminescent substrate (New England BioLabs), according to manufacturer's instructions and exposed to X-ray film.

### Adherence of Bacterial Cells to Immobilized Recombinant Human Cytokeratin 10

Adherence of S. aureus to immobilized recombinant human cytokeratin 10 (rMK10) was performed as follows. Nunc-Immuno MaxiSorb microtitre plates were coated with rMK10 in carbonate buffer (15 mM Na_2_CO_3_, 35 mM NaHCO_3_ [pH 9.6]) and incubated overnight at 4 °C. Bovine serum albumin (5 mg/ml) was added, and the plates were incubated for 2 h at 37 °C. The plates were washed three times with PBS. A bacterial cell suspension (OD_600_ = 1.0 in PBS) was added (100 μl/well), and the plates were incubated for 1.5 h at 37 °C. Plates were washed three times with PBS; bound cells were fixed with formaldehyde (25% v/v) for 30 min and then stained with Crystal Violet (0.5% v/v, 100 μl/well) for 1 min. After three washes with PBS, acetic acid (5% v/v) was added (100 μl/well), and cells were incubated for 10 min at room temperature. The absorbance was measured at 570 nm in an ELISA plate reader (Labsystems Multiskan Plus).

### Microbiological Procedures

Nasal specimens were obtained with sterile cotton swabs (Tran swab; Medical Wire & Equipment) from each nostril separately. Swabs were processed immediately by vortexing vigorously in Stuart's medium. Dilutions of this medium were inoculated on phenol red-mannitol salt agar (PHMA) and in phenol red-mannitol salt broth (PHMB) according to previously published protocols for quantitative carriage assessment [18,26]. All positive postinoculation cultures for S. aureus were screened for tetracycline resistance, by plating on media with and without tetracycline, to assess the presence of tetracycline-resistant S. aureus strains in the endogenous nasal flora of the participant. The DU5997 S. aureus strain has low-level tetracycline resistance (minimal inhibitory concentration: 2 μg/ml). Technicians reading the agar plates were unaware what strains were inoculated in the volunteers (blinded assessment).

### Bacterial Genotyping

Testing for tetracycline resistance was performed by disk diffusion. Resistance was verified by PCR with specific primers spanning the insertion site of the tetracycline resistance cassette. Furthermore, two random isolates per culture (either resistant or sensitive to tetracycline) were genotyped by pulsed-field gel electrophoresis (PFGE) (CHEF Mapper; Biorad). DNA within the plugs was digested using 20 U SmaI (Roche Molecular Diagnostics) during 4 h at 25 °C in SuRe-cut buffer. The electrophoresis protocol was performed at 6 V/cm and 14 °C using 0.5× TBE buffer (Biorad). The program consisted of two blocks: 10 h at 60°/−60° angles with a switch time of 5–15 s, followed by 10 h at 60°/−60° angles and a switch time of 15–45 s. Strains were considered to be clonally related if their PFGE fingerprints did not differ by more than three bands [27].

### Anti-ClfB ELISA

The working volume of serum for the ELISA was 100 μl. After each step, the microplates were washed three times with phosphate-buffered saline (PBS; pH 7.4) containing 0.1% v/v Tween 20. The microplates were coated with recombinant ClfB in 50 mM sodium carbonate (pH 9.5) overnight at 4 °C. Blocking was performed with 5% (w/v) bovine serum albumin in PBS for 2 h at 37 °C and washing for three times with PBS. Serum of the volunteers was then applied and incubated for 1 h at room temperature with shaking. Dilute (1:10,000) horseradish peroxidase conjugated to rabbit anti-human antibodies (Dako) was then added to the wells, and the incubation was continued for 1 h at room temperature. The reaction was developed by the addition of tetramethylbenzidine and stopped by adding H_2_SO_4_. The reaction was measured at 450 nm in an ELISA plate reader (Bio-Rad).

### Sample-Size Calculation

An earlier inoculation study showed that after artificial inoculation, more than half of those inoculated still carried the inoculating strain at 1-mo follow-up [18]. We estimated that at the end of follow-up, the 8325–4 strain would still be present in 50% of the inoculated volunteers, and the DU5997 strain in 10%. With a confidence level of 95% and a power of 80%, we would need to inoculate both the 8325–4 strain and the DU5997 strain in at least 15 healthy volunteers.

### Statistics

The primary outcome measurement was the survival time of both the 8325–4 and DU5997 strain per individual, and analyzed by Kaplan-Meier survival analysis (log-rank test). The survival time was defined as the number of days until the last positive culture. S. aureus strains still present at the end of follow-up were censored. The mean numbers of 8325–4 versus DU5997 S. aureus cfu were compared by two-way ANOVA, after log transformation of the number of cfu. Other comparisons of means were performed by *t*-test, Mann-Whitney *U* test, or one-way ANOVA, where appropriate. Nonparametric correlations were estimated for ClfB antibody titer and time to S. aureus clearance from the nose. *p*-Values, two-tailed, below 0.05 were considered statistically significant. All statistical analyses were performed with SPSS 11.5 for Windows software (SPSS). Means and medians are given with their standard deviations or interquartile range.

## Results

### Nasal Carriage Status before Inoculation

None of the volunteers included in the present study encountered adverse effects, and all adhered to the study protocol for the duration of the experiment. At the start of the protocol, the carriage status was assessed: there were six persistent carriers, two intermittent carriers, and eight non-carriers. All volunteers received mupirocin nasal ointment 5 wk before inoculation (Figure 1). Five volunteers still carried low levels of S. aureus in their noses after mupirocin treatment, just before inoculation (median: 1 cfu/ml). The endogenous S. aureus strains of the volunteers who were carriers had distinct PFGE fingerprints compared to the test strains (unpublished data).

### The DU5997 Strain Has the Same Fitness as the 8325–4 Strain

**Figure 2:**
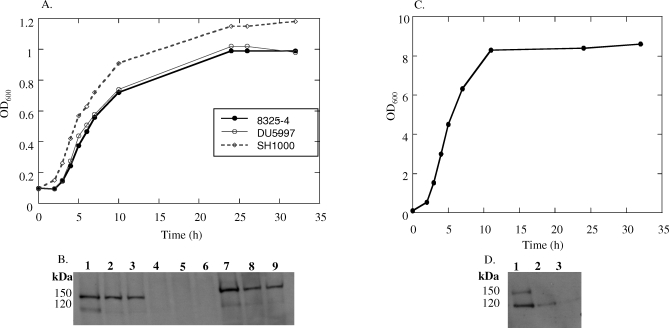
ClfB Expression Experiments (A) Bacterial growth and ClfB expression in RPMI. Strains 8325–4, DU5997, and SH1000 were grown in RPMI, and samples were taken at regular intervals to monitor growth. (B) Samples taken at early exponential, early stationary, and late stationary phases of growth were analyzed by Western immunoblotting. Lanes 1, 2, and 3 show ClfB expression from strain 8325–4 at early exponential, early stationary, and late stationary growth phases, respectively. Lanes 4, 5, and 6 show ClfB expression from strain DU5997 at early exponential, early stationary, and late stationary growth phases, respectively. Lanes 7, 8, and 9 show ClfB expression from SH1000 at early exponential, early stationary, and late stationary growth phases, respectively. (C) Bacterial growth and ClfB expression in TSB. Strain 8325–4 was grown in TSB, and samples were taken at regular intervals to monitor growth. (D) Western immunoblotting was carried out to detect ClfB expression at early exponential, early stationary, and late stationary phases of growth. Lanes 1, 2, and 3 show ClfB expression from strain 8325–4 grown in TSB at early exponential, early stationary, and late stationary growth phases, respectively.

We compared the growth of strain 8325–4 with SH1000 (which has a fully functional SigB), and with DU5997 (which is defective in ClfB). Growth was carried out in a medium reflective of growth conditions in vivo. The results show that bacteria grew more slowly and to a much lower optical density in stationary phase in RPMI as compared to TSB. Doubling times for 8325–4 were 108 min in RPMI and 40 min in TSB. In RPMI, the growth rates and yields of 8325–4 and DU5997 were indistinguishable, whereas SH1000 grew slightly faster (doubling time 96 min) and to a higher density (Figure 2A).

### ClfB Expression-Dependent Adherence to Cytokeratin 10

Expression of ClfB by RPMI- and TSB-grown bacteria was compared. Previously, it was shown that ClfB protein was not detectable on 8325–4 cells from the stationary phase of growth in rich media, and this was confirmed here (Figure 2B) [28]. In contrast, intact ClfB was present at the same level in RPMI-grown SH1000 and 8325–4 during all phases of growth, including late stationary phase (Figure 2B).

**Figure 3:**
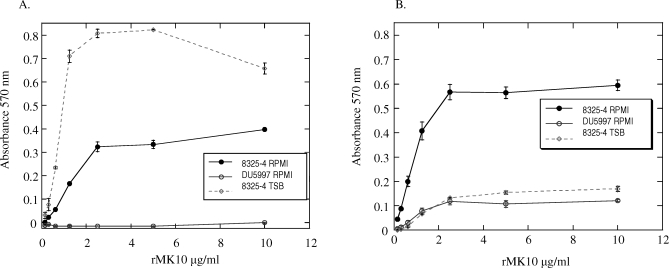
S. aureus Adherence to Recombinant Cytokeratin 10 in Different Growth Stages Data are epresentative of three independent experiments. Bars represent standard deviation. (A) Adherence of bacterial cells in exponential phase of growth to rMK10. Strain 8325–4 was grown to exponential phase in RPMI or TSB, and its ability to bind rMK10 was compared to DU5997 grown in RPMI. (B) Adherence of bacterial cells in stationary phase of growth to rMK10. Strain 8325–4 was grown to stationary phase in RPMI or TSB, and its ability to bind rMK10 was compared to DU5997 grown in RPMI.

Bacterial cells grown to mid-exponential phase were tested for their ability to adhere to immobilized CK10 (Figure 3). Strain 8325–4 grown in TSB adhered more strongly to CK10 than 8325–4 grown in RPMI, despite degradation of some ClfB molecules to the nonfunctional form lacking domain N1 [28]. DU5997, which is completely defective in ClfB expression, adhered poorly, indicating that the adherence of RPMI-grown bacteria to CK10 is dependent on the presence of ClfB.

In contrast to exponential phase cells, 8325–4 grown to stationary phase in TSB was defective in adherence (Figure 3), which is consistent with the lack of ClfB shown in Figure 2D. Strain 8325–4 grown to stationary phase in RPMI adhered more strongly to CK10 than the equivalent number of cells from exponential phase, whereas the DU5997 bound poorly.

### Strain 8325–4 Persists in the Nose, DU5997 Does Not

Upon inoculation of 8325–4 and DU5997 *ClfB^−^* cells, the majority of the volunteers (14/16) did not harbor the DU5997 strain after day 4, whereas fewer volunteers (7/16) lacked the 8325–4 strain after this stage. After 2 wk, all volunteers had eliminated the DU5997 strain, whereas the 8325–4 strain was still present in three volunteers after 28 d, at the end of follow-up. These three volunteers were found to be persistent or intermittent nasal carriers during the preinoculation screening.

**Figure 4:**
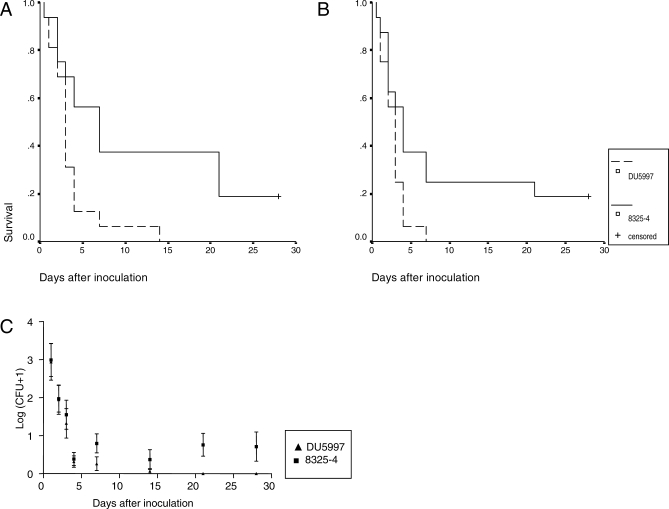
Survival in the Nose of the Wild-Type Strain versus the Mutant Strain (A) Kaplan-Meier survival curve of the 8325–4 S. aureus (solid line) and its *ClfB^−^* mutant (DU5997) strain (dashed line) in human nose (last cultured strain in either nostril). All cells of the mutant strain were eliminated after a period of 14 d, which is significantly faster than the elimination rate for the 8325–4 S. aureus strain. (B) Kaplan-Meier survival curve of the 8325–4 S. aureus (WT) and *ClfB^−^* mutant (DU5997; dashed line) that were inoculated together as a mix in one nostril. (C) Number of cfu of 8325–4 and DU5997 in follow-up samples.

The overall median survival time of the 8325–4 strain was significantly higher than the DU5997 strain: a median of 7.0 (95% confidence interval [CI], ±4 d; interquartile range: 2–21 d) compared to 3 (95% CI, ±1 d; interquartile range: 2–4 d; log-rank: *p* = 0.006; Figure 4A). The viable counts of S. aureus after inoculation were always higher for the 8325–4 strain (Figure 4C), but not significantly. Inoculated S. aureus cells could be cultured for a longer mean follow-up period from intermittent carriers (18.3 ± 12.1 d) than from persistent (7.0 ± 8.6 d) or non-carriers (5.4 ± 6.3 d; one-way ANOVA: *p* = 0.025). None of the volunteers used antibiotics during the follow-up period. Identification of the inoculation strains was confirmed by tetracycline susceptibility testing, PFGE, and PCR analysis of the *clfB* locus.

Analyzing the survival within the nostril in which the mix was inoculated also shows that the 8325–4 strain survives significantly longer: a median of 4.0 (95% CI, ± 1 d; interquartile range: 2–7 d) compared to 3.0 (95% CI, ±1 d; interquartile range: 1–3 d; log rank: *p* = 0.033; Figure 4B). Comparing the survival of the 8325–4 strain inoculated singularly in eight nostrils versus the DU5997 strain inoculated in eight other volunteers, also showed a longer survival for 8325–4, but this did not reach statistical significance (unpublished data). In this experiment, we also evaluated whether strains crossed over to the contralateral nostril, irrespective of bacterial load and strain displacement. In two instances, there was crossover from the DU5997 strain from the mix to the contralateral nostril where only 8325–4 strain was inoculated (only on the first day after artificial inoculation). The 8325–4 strain from the mix crossed over in six volunteers to the contralateral nostril where only the DU5997 was inoculated, occurring as of day 1 up to day 28 of follow-up.

### Dynamics in ClfB Antibody Response

To assess the role of the anti-ClfB antibody response in the survival of the inoculating strains, we performed anti-ClfB ELISA. The anti-ClfB antibody levels in volunteers did not change during the 4 wk following inoculation. However, volunteers who were carriers of S. aureus had a higher anti-ClfB antibody level: 2.0 ± 0.7 (extinction), than those who were non-carriers: 1.3 ± 0.7 (Mann-Whitney *U*: *p* = 0.093). Those volunteers who were still carrying the 8325–4 strain after 28 d also had a higher anti-ClfB antibody titer compared to those who were free of S. aureus: 2.4 ± 0.4 versus 1.5 ± 0.7 (Mann-Whitney *U*: *p* = 0.069). Finally, there was no correlation between the anti-ClfB antibody titer and the time needed to eradicate S. aureus from the nose (unpublished data).

## Discussion

We report here the first study, to our knowledge, of healthy human volunteers being coinoculated with an S. aureus 8325–4 strain and an isogenic mutant defective in the MSCRAMM ClfB (DU5997). We show that ClfB is an important factor for establishing human nasal colonization by S. aureus, as the *ClfB^−^* mutant (DU5997) was not able to survive in the human nose after 2 wk. In contrast, the 8325–4 strain was still present at the end of follow-up in three volunteers.

Because we performed two different inoculation protocols within each volunteer, crossover of strains to the contralateral nostril complicated the analysis of this study. The 8325–4 strain from the mix crossed over in six volunteers, whereas the DU5997 strain only crossed over in two instances. Furthermore, the 8325–4 strain showed this phenomenon up to the last day of follow-up. The DU5997 only crossed over on the first day of follow up and never after. These findings highlight the complexity of interpretation of in vivo studies but strongly suggest that 8325–4 is better able to colonize than DU5997.

The results of our study regarding the number of volunteers still colonized after 1 mo are different than the assumptions of our sample-size calculation. At the end of follow-up, only 19% of the volunteers carried the 8325–4 strain, not 50%. This low carriage may be due to the fact that we inoculated only one type of S. aureus strain and not a mix with different clones as performed by Nouwen et al. [18]. In addition, the 8325–4 strain has been propagated in the laboratory for a long period and might have lost most of its capacity to effectively colonize humans. On the other hand, the survival of the *ClfB^−^* mutant (DU5997) was lower than estimated (0% at day 28, instead of 10%), illustrating the crucial role of ClfB in adherence to the nasal mucosa.

Interestingly, 8325–4 grown to stationary phase in a nutrient-deficient medium, comparable to that of the nasal niche, adhered to rK10, whereas the same strain grown in a rich medium did not. Thus, it seems plausible that ClfB is expressed in all S. aureus growth stages in the nose, a relatively nutrient-deficient environment. Expression of the *clfB* gene directly in the growth environment of the human nose has been demonstrated previously [10]. Furthermore, the *ClfB^−^* mutant grown in nutrient-deficient media was unable to adhere to immobilized CK10. These in vitro study results support our in vivo findings.

Other in vitro studies showed ClfB-deficient mutants could still adhere to nasal epithelial cells [10]. Even though these experiments were not performed in nutrient-deficient media, it is unlikely that ClfB is the sole determinant of nasal colonization. Other S. aureus surface factors, including capsular polysaccharides, teichoic acid, and iron-responsive surface determinant protein (IsdA), also promote adherence to the nasal mucosal surfaces [15,29,30]. These studies were performed either in vitro or in animals. Clearly, S. aureus adherence to and colonization of the epithelium in the nares is complex and multifactorial. Only the direct application of viable, genetically modified S. aureus cells in their natural niche, the human vestibulum nasi, will allow functional characterization of both human and microbial factors required for colonization.

Regarding the complexity of the human factor: in this study. the anti-ClfB antibody response was also assessed. No rise in the anti-ClfB response after the artificial colonization was documented. This may be explained by the fact that 8325–4 has a defect in the stationary phase SigB and is therefore considered less virulent, and may therefore be less “immunogenic.” We did find that, in general, S. aureus carriers had higher anti-ClfB levels than non-carriers. This is concordant with the results of Dryla et al. [31], who found significantly higher IgG levels in S. aureus nasal carriers.

Effective intervention in S. aureus colonization for the prevention of subsequent infection requires the identification of human and bacterial factors important in establishing nasal colonization [6,7]. This study of a 8325–4 strain and an isogenic *ClfB^−^* null mutant revealed the relevance of the staphylococcal adhesin ClfB in the colonization of the human nose and demonstrates that ClfB is a potential target for S. aureus decolonization strategies.

### Limitations of This Study

A limitation of this study was the use of the 8325–4 strain. As mentioned before, this strain has a defect in the stationary phase SigB and is therefore unlikely to colonize and persist as well as wild-type strains we would find in natural S. aureus nasal carriers. We chose this strain because it facilitated a safe inoculation protocol for humans and actually made this experiment possible. A S. aureus strain from a natural carrier with no major virulence factors, such as superantigens, would be preferred for future artificial inoculation experiments.

## Abbreviations

cfu: colony-forming unit
CI: confidence interval
CK10: cytokeratin 10
ClfB: clumping factor B
MSCRAMM: microbial surface components recognizing adhesive matrix molecule
PBS: phosphate-buffered saline
PFGE: pulsed-field gel electrophoresis
SigB: sigma factor
TSB: tryptic soy broth
